# Changing times? Gender roles and relationships in maternal, newborn and child health in Malawi

**DOI:** 10.1186/s12884-017-1523-1

**Published:** 2017-09-25

**Authors:** Lucinda Manda-Taylor, Daniel Mwale, Tamara Phiri, Aisling Walsh, Anne Matthews, Ruairi Brugha, Victor Mwapasa, Elaine Byrne

**Affiliations:** 1 University of Malawi, College of Medicine, Blantyre, Malawi; 2 Royal College of Surgeons in Ireland, Dublin, Ireland; 3 Dublin City University, Dublin, Ireland

**Keywords:** Pregnancy, Maternal and child health, Male involvement, Antenatal care, Community systems, Health systems, Access, Barriers, Malawi

## Abstract

**Background:**

For years, Malawi remained at the bottom of league tables on maternal, neonatal and child health. Although maternal mortality ratios have reduced and significant progress has been made in reducing neonatal morality, many challenges in achieving universal access to maternal, newborn and child health care still exist in Malawi. In Malawi, there is still minimal, though increasing, male involvement in ANC/PMTCT/MNCH services, but little understanding of why this is the case. The aim of this paper is to explore the role and involvement of men in MNCH services, as part of the broader understanding of those community system factors.

**Methods:**

This paper draws on the qualitative data collected in two districts in Malawi to explore the role and involvement of men across the MNCH continuum of care, with a focus on understanding the community systems barriers and enablers to male involvement. A total of 85 IDIs and 20 FGDs were conducted from August 2014 to January 2015. Semi-structure interview guides were used to guide the discussion and a thematic analysis approach was used for data analysis.

**Results:**

Policy changes and community and health care provider initiatives stimulated men to get involved in the health of their female partners and children. The informal bylaws, the health care provider strategies and NGO initiatives created an enabling environment to support ANC and delivery service utilisation in Malawi. However, traditional gender roles in the home and the male ‘unfriendly’ health facility environments still present challenges to male involvement.

**Conclusion:**

Traditional notions of men as decision makers and socio-cultural views on maternal health present challenges to male involvement in MNCH programs. Health care provider initiatives need to be sensitive and mindful of gender roles and relations by, for example, creating gender inclusive programs and spaces that aim at reducing perceptions of barriers to male involvement in MNCH services so that programs and spaces that are aimed at involving men are designed to welcome men as full partners in the overall goals for improving maternal, neonatal and child health outcomes.

**Electronic supplementary material:**

The online version of this article (10.1186/s12884-017-1523-1) contains supplementary material, which is available to authorized users.

## Background

The United Nations Task Force on Child and Maternal Health recommends that the highest priority be given to strengthening the primary healthcare system, from community-based interventions to the first referral-level facility at which emergency obstetric care is available [1]. This recommendation is echoed in the 2007 Road Map for accelerating Maternal and Newborn Health in Malawi, which articulates the following two objectives: firstly, to increase availability, access, utilization of quality skilled obstetric care during pregnancy, childbirth and post natal care at all levels of the health care delivery system, and secondly, to strengthen the capacity of individuals, families, communities, civil society organizations and government to improve maternal health [2]. The emphasis on encouraging and supporting individuals, families, communities and civil society organizations to participate in maternal and newborn health comes from the recognition that maternal mortality and newborn survival can be reduced substantially through community or home-based initiatives [3–5].

Recent global trends in the delivery of maternal and child health services have been to move as many of those services as close to the community as possible [6]. Most obstetric complications occur unexpectedly around the time of delivery in women with no risk factors, which means that removing any potential obstacles that communities and individuals may experience in accessing health facilities is key [1]. Improved access not only depends on the availability of health services, but also on community factors that influence the decision-making of individuals. There is widespread recognition that involving men in maternal and child health services offers positive benefits [1]. Numerous studies have demonstrated that male involvement can have a positive impact on the utilization of maternal, newborn and child health (MNCH) services [7–11].

One area of published literature on male involvement in MNCH examines strategies on engaging men in HIV treatment and prevention strategies in the context of antenatal care (ANC) services. For example, there is evidence that in Maharashtra in India male partners can significantly impact women’s uptake of HIV-related services and support adherence to antiretroviral drug regimens, especially in the context of ANC services [12]. Likewise, in Kenya, partner participation in HIV Counselling and Testing (HCT) and couples counselling not only increased partner attendance to 15%, but also increased Nevirapine and formula feeding uptake among HIV positive women attending antenatal clinics [13]. Furthermore, women who disclose their HIV status to their partners have a higher likelihood of accepting ARV prophylaxis, adhering to feeding choices and carrying out safer sex practices, unlike women who lack partner support [8, 14–17]. Several studies found that lack of male involvement can impact negatively on access and utilisation of services. For example, the majority of women in a number of studies refused HIV testing in Prevention of Mother to Child Transmission (PMTCT) settings because their partners had either not been present or had not given their permission to test [18–20]. While efforts to involve men have been addressed in the context of ANC/PMTCT this is only one component of addressing the health needs of women of reproductive age, newborns and children under-5 years of age.

Another area of published literature highlights the influence and implications male partners have more broadly in MNCH service utilisation. For example, Aarnio, Chipeta and Kulmala state that male partners in most cultures are the main decision makers in families; hence men affect women’s health care seeking behaviour, such as the utilisation of ANC, family planning services, or the preferred institution for delivery [21]. A study in Ghana refers to the above as gatekeeping whereby compound heads and husbands who control the economic resources and household decisions impede women’s prompt access to health care services [22]. Other studies in Uganda, Burkina Faso, Nigeria and Bangladesh echo the same dichotomous tension by highlighting the important role men play in their families and communities, which can support or compromise women’s sense of autonomy and affect women’s health seeking behaviour patterns [23].

Despite evidence that male partner support can increase women’s uptake of maternal and child services, few men are reported as participating in MNCH programmes [15, 23]. Even when men want to be involved in maternity care, they are often discouraged from doing so because of societal and health system norms [24]. When men do get involved they can feel uncomfortable [24] particularly in confined or limited “spaces” at health facilities that have been traditionally reserved for women. There is therefore a contradiction between men’s positive attitudes towards male involvement in MNCH and their actual low participation rates suggesting that external barriers play a large role in getting men involved [25]. It is important, therefore, to recognize the role of gender norms as determinants of male involvement and as a potential source of stress for men striving to adhere to multiple roles [21].

### Gender constructions and male involvement in Malawi

The construction of masculinity in patriarchal societies often limits the ways in which men are “allowed” to engage in pregnancy, birth and child rearing [26]. In many patriarchal societies, men still have the final say on issues related to family planning, reproductive health, their wives’ and daughters’ labour market participation, and the use of family resources, including medical and educational expenditures [27]. Malawian society is largely male-dominated, even with regard to female reproductive health [28]. In Malawi, pregnancy and childbirth has traditionally been a woman’s domain and maternal health care services have focused on women, with very little attention paid to male involvement [29]. In addition, in Malawi, as Kululanga et al. observe, women have been supported by other women during labour and birth [30]. Furthermore, the notion of male involvement during labour and birth is perceived as a foreign culture not commonly practiced in Malawi [31]. As such, programs and policies that seek to involve men in MNCH is a relatively new approach in Malawi [29]. However, because global trends recognise that men’s involvement in maternal and child health is a key driving force in improving MNCH access and utilisation the Malawi government’s policy document for accelerating the reduction of MNCH mortality and morbidity specifically articulates the need to empower communities, especially men, to contribute to timely referrals [2]. In addition, the Malawi National Sexual and Reproductive Health and Rights (SRHR) policy describes the need for male involvement in the development, promotion and delivery of SRHR in the context of maternal and neonatal health [32].

These policy documents have spurred the exploration and implementation of a number of strategies and programs to engage men in sexual and reproductive health programs in Malawi. For example, one study revealed the usefulness of an invitation card during pregnancy as a strategy to enhance male partner involvement in PMTCT services [33]. Another study, the Malawi Male Motivator Project, examined the role of peer educators as a strategy to engage men as partners in order to promote better sexual, reproductive and maternal health outcomes. Through the use of male peers in the community, the study reported men’s increased understanding of and ability to discuss contraception and family planning [34]. In addition, a key program that encourages involving men in antenatal care to increase women’s uptake of antenatal care services is the Safe Motherhood initiative. Under the Safe Motherhood project, women and men receive nutrition information and guidance at clinics [35]. Further, men receive advice on how to support their partners during pregnancy. Most importantly, the Safe Motherhood initiative in Malawi has empowered traditional leaders to make sure that all pregnant women deliver at a health facility [36]. This intervention is part of strategy number seven in the Road Map, which aims at empowering communities to ensure continuum of care between the household and health care facility [2].

The MNCH continuum of care is the operational context for health programming to ensure that there is continuity of care for women and children across the Reproductive Maternal Newborn and Child Health (RMNCH) services [37]. The first dimension of the continuum of care is the time from pre-pregnancy, through pregnancy, childbirth, and the early days and years of life [38]. The second dimension links the various levels of home, community and outreach, and health facilities [38]. The impact of the MNCH continuum of care programs depends on (1) high coverage of essential interventions throughout the continuum; (2) their quality; and (3) functional linkages between interventions and the health system [37].

The recently published Malawi Demographic and Health Survey (2015–2016) shows some improvements in key areas that point to the increasing uptake in maternal health services. For instance, the MDHS shows that most mothers (95%) received ANC services from a skilled health care provider [39]. Additionally, the proportion of women who received ANC in the first trimester has more than doubled from 9% in 1992 to 24% in 2015–2015 [39]. Moreover, institutional deliveries have increased from 55% in 1992 to 91% in 2015–2016 and over the same time period, home deliveries have decreased from 43% in 1992 to 7% in 2015–2016 [39].

In spite of the progress in Malawi, there are still some notable gaps. For example, the proportion of women (15–49) that received four or more ANC visits generally declined from 1992 (62%) to 2010 (46%) before rising modestly to 51% [39]. Furthermore, the 2015–2016 MDHS found that among women aged 15–49 giving birth in the 2 years before the survey, 42% had a postnatal check during the first 2 days after birth. Half of mothers (50%) did not receive postnatal check [39].

The above information provides an opportunity for reflection on male involvement in maternal, newborn and child health in Malawi. In this paper, male involvement refers to the actions and decisions men make at a community level to that either promote or hinder women’s utilization of MNCH services.

## Objectives

The research presented in this article was a component of a Community Systems Strengthening for Equitable Maternal, Newborn and Child Health (COSYST-MNCH) project in Malawi. The overall goal of the COSYST-MNCH project was to achieve a better understanding of community systems factors underpinning maternal, newborn and child health (MNCH) services in Malawi, focusing on the health dimensions of the first 1000 days of life. In Malawi, there is still minimal, though increasing, male involvement in ANC/PMTCT/MNCH services, but little understanding of why this is the case. The aim of this paper is to specifically explore the role and involvement of men in MNCH services, as part of the broader understanding of those community system factors.

## Methods

A mixed method convergent research design within a transformative paradigm (that is seeking sustainable changes in behavior and systems to improve social development) was adopted for the COSYST-MNCH project [40]. The aim was to collect data, quantitative and qualitative, that would enable the identification and a better understanding of what community systems exist and how they function. This paper draws on the qualitative data collected in two districts (Mchinji and Nkhotakota) in Malawi to explore the role and involvement of men across the MNCH continuum of care, with a focus on understanding the community systems barriers and enablers to male involvement in the promotion of MNCH.

## Setting

Two rural districts in Malawi were selected as case studies. A case study is a research strategy that involves an in-depth empirical investigation of a phenomenon within its real life context using a variety of data collection methods [41, 42]. The first phase of the research consisted of the selection of communities for the case studies. Two Traditional Authorities (T/As) in Mchinji (Mduwa and Mkanda) and Nkhotakota (Mwadzama and Malengachanzi) districts were selected. The main criteria for communities to be selected were that (i) the non-governmental organization (NGO) partner - Concern Worldwide had current (or recently completed) implemented projects/interventions; and (ii) that the NGO partner had established links with other stakeholders working in these districts. In addition, the District Health Officials (DHOs) in each of the two selected districts advised the researchers that the research participants would be able to provide valuable information and feedback on the enablers and barriers to MNCH service utilisation because of the distances that some of these villages have in terms of location to health centres and other health care services and facilities.

Health Surveillance Assistants (HSAs) in the two districts facilitated with identifying community members who were willing to participate in the research. This process required community sensitization and engagement in order to obtain project support, for identifying potential respondents in the community and to ensure good contextual understanding and knowledge of the communities and on-going activities. This was important for gaining entry into the communities.

The third stage involved conducting in-depth interviews (IDIs) and focus group discussions (FGDs) (refer to Additional files 1, 2, 3, 4 and 5), in each of the four selected Traditional Authorities. Data were collected from October 2014 to January 2015 by two Malawian research assistants (1 male and 1 female) who were trained on the protocol and data collection methods and conducted the interviews and focus group discussions. The research assistants were familiar with the local context, spoke the local language (Chichewa) and were not associated with the NGO operating in the traditional authorities. A female Malawian project coordinator provided local supervision.

**Fig. 1:**
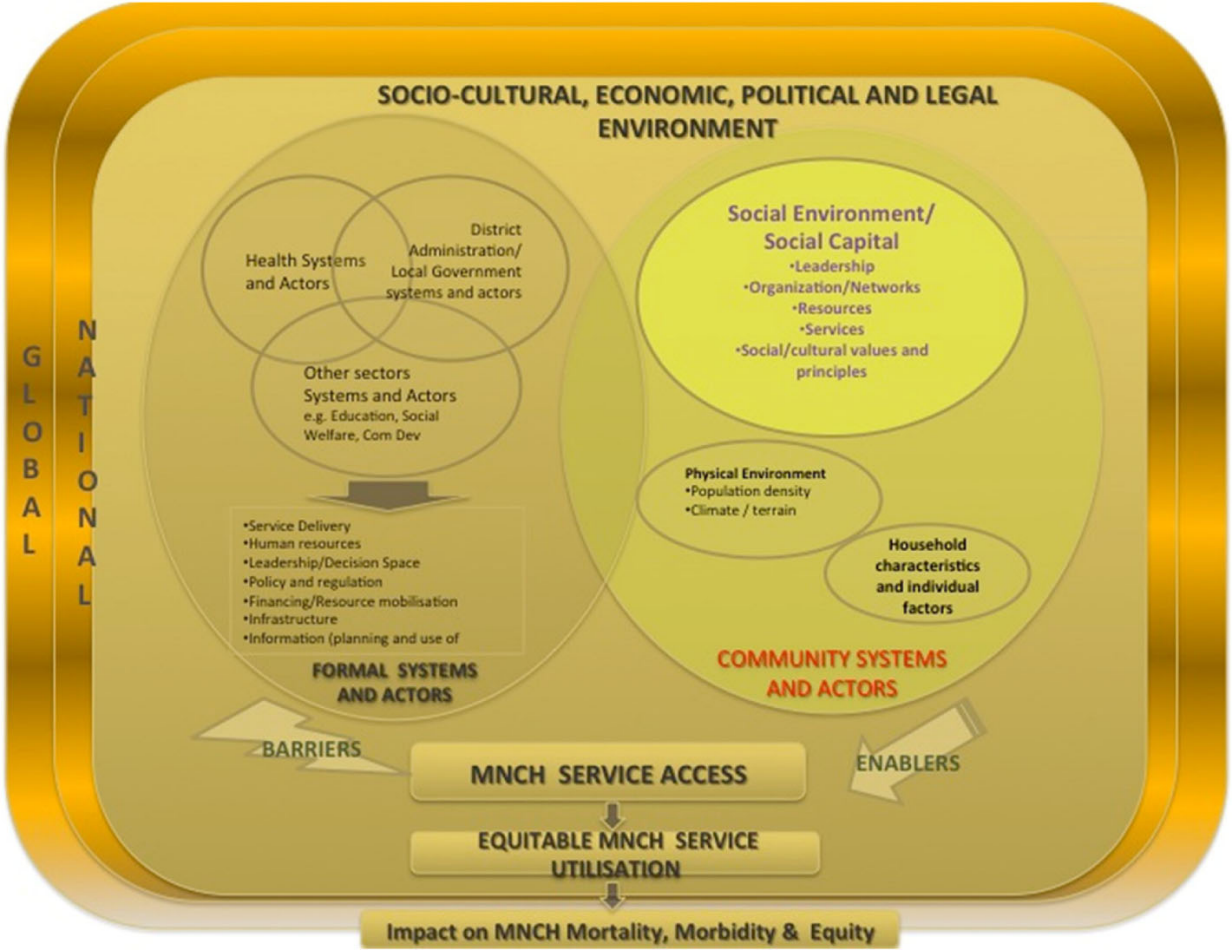
COSYST-MNCH community systems analytical framework

A document review of existing literature on community systems and health systems, and a COSYST Community Systems Analytical Framework was drafted for analysing and describing community system enablers and barriers that explain the utilization of MNCH services. The framework, depicted in Fig. 1 below, informed the design of the interview guides. The broad ideas explored the formal and informal community systems enablers and constraining factors to MNCH service utilisation whereby a core set of questions focused on utilisation of specific MNCH services for example, ANC, delivery or PNC.

Prior to data collection, the questions were read for familiarization and to identify questions that were ambiguous or irrelevant; these were revised or removed. The tools were pretested/piloted in the two districts selected in order to check for clarity, relevance, comprehensiveness and flow of questions. Once again, questions that were identified as ambiguous or not relevant to answering the main study objectives were altered or omitted.

Interviews and FGD were held in private spaces. For instance, the FGDs were conducted in school halls or local churches and the IDIs were conducted in district health offices, private rooms in the health centres and at people’s residences in the community. The FGDs comprised of a homogenous group of individuals in the following categories: women of child-bearing age who are using/not using MNCH services, and those affected by disease or vulnerabilities and household members such as husbands and grandmothers, mothers in law. Each FGD had 6–12 participants. The IDIs comprised of senior representatives of NGO and government departments units, District Assembly/Commission, and members of the District Health Management Team. We also interviewed village chiefs and religious leaders, traditional healers, nurses, HSAs, clinical officers and women who were utilizing MNCH services. A total of 85 IDIs and 20 FGDs were conducted. Tables 1 and 2 show the attributes of participants.

**Table 1:** In-depth interviews conducted

	MCHINJI							NKHOTAKOTA						
	Mduwa		Mkanda		Mchinji District Hospital		NUMBER	Malengachanzi		Mwadzama		Nkhotakota District Hospital		NUMBER
	M	F	M	F	M	F		M	F	M	F	M	F	
Traditional Leaders	6		2				8	3		6				9
Religious Leaders	5		2				7	4		3				7
Government Officials					4	1	5					3	2	5
Senior NGO officials					1	7	8				1	5	2	8
Health personnel	2	1		2			5	2		1	2			5
Health Surveillance Assistants	2		1				3	2	3	4				9
Traditional Birth Attendants		1		2			3		2		1			3
TOTAL	15	2	5	4	5	8	39	11	5	14	4	8	4	46

**Table 2:** Focus group discussions conducted

	MCHINJI					NKHOTAKOTA				
	Mduwa		Mkanda			Malengachanzi		Mwadzama		
	M	F	M	F	NUMBER	M	F	M	F	NUMBER
Caregiver-Husbands	1		1		2	2		1		3
Caregiver-Grandmothers + Mother in-law		2			2		1			1
MNCH users (pregnant women + women with under-5 children)		2		2	4		1		2	3
MNCH non-users (pregnant women + women with under-5 children)		1		1	2		1		2	3
TOTAL	1	5	1	3	10	2	3	1	4	10

Participants were purposively sampled based on their socio-demographic characteristics including, age, occupation, parity, roles education and experiences. A diverse mix of educated (primary, secondary or tertiary) and uneducated (never been to school), singe, married, divorced and widowed men and women were interviewed whose occupations ranged from working as a manager for an NGO, in the hospital as a nurse, clinical officer or HSA to farming and owning small businesses in the community. The religious affiliations of the participants were Christian or Muslim. The community and religious leaders interviewed were all men. Traditional birth attendants (TBAs) were all women.

However, snowball sampling was also used to encourage interviewees to nominate other participants who met the eligibility criteria, but were hard-to-reach. In our study, snowball sampling was used to identify and interview TBAs and women of child-bearing age not using MNCH services as also asking interviewees to nominate other people they believed would have important contributions to make to the research. The IDIs and FGDs were conducted in Chichewa (the local language), audio-recorded (with permission) and later transcribed in Chichewa and then translated to English. Interviews continued until saturation was reached. The research assistants and the project coordinator checked the transcripts for completeness against the recorded interviews.

## Data analysis

Data analysis was iterative, for instance, on-going throughout the data collection process. A point of saturation approach was also applied to the total sample of healthcare workers, local opinion leaders (traditional and religious), senior government and non-governmental officials, users and non-users of MNCH services and the relatives of mothers (husbands and grandmothers). Given that data analysis was iterative, saturation was achieved when no more patterns or themes emerged from the data. Once all the data had been gathered, translated and transcribed the Malawian and Irish researchers’ coded data separately. The involvement of two separate coding teams experienced in qualitative research methods demonstrates the rigor, which was applied to the analysis and evaluation of the data. Each team presented key themes and sub-themes and a joint meeting agreed upon a final coding scheme for all the data collected.

Thematic analysis was the data analysis technique used to identify the main themes generated from the research. Thematic analysis was preferred because it best fit the search for evidence on the barriers and enablers to MNCH service utilization. Specific themes corresponding with the study objectives were immediately identified while additional themes emerged during the analytical process. Using NVivo 10, codes were developed from the responses and grouped into themes and sub-themes. The main themes were in the category of parent nodes and the sub-themes were in the category of child nodes. The main theme that is developed in this paper was that on male involvement. We used the structure of our interview guides, which we had based our literature review, to highlight what appeared to be system enablers and barriers to accessing MNCH services.

## Ethical approval

The University of Malawi’s College of Medicine Research and Ethics Committee (COMREC No granted ethical approval for the research. P.08/13/1443). In addition, permission to collect data in Mchinji and Nkhotakota was obtained from the Ministry of Health’s Reproductive Health Unit. Written informed consent was obtained from individual participants. Literate participants provided a signature on the consent form, and illiterate participants provided a thumbprint. Interviews were conducted in a private space and participants were assured that their personal details would be omitted from transcripts to ensure confidentiality. Participants were also informed that any information generated by the research might be published, but that confidentiality would be maintained and no personal details would be divulged. Lastly, participants were informed that their involvement in the research was voluntary and that withdrawal was permitted at any time and without personal consequence.

## Results

Within the context of community systems and actors, the main finding here was on male involvement in MNCH service utilization. Our study generated three main themes under male involvement that describe community systems and actors that enable or hinder women’s utilization of MNCH services. In the context of this research, barriers are understood as any factors or reasons that impede or hinder women’s utilization of MNCH services. Conversely, enablers are understood as factors or reasons that facilitate or support women’s utilization of MNCH services. The themes identified to describe community system enablers and barriers of MNCH service utilisation in Mchinji and Nkhotakota were identified using the framework depicted in Fig. 1, above. Our three main specific themes included traditional gender roles at home; policy-changes to improve male involvement in MNCH, and; community/local initiatives to reinforce male involvement that operate within the socio-cultural milieu.

### Gender roles at home

Barriers to MNCH utilisation were identified at household and facility levels. In spite of these obstacles, there was general agreement on the importance of male involvement in seeking healthcare and services for their families in particular in the decisions to attend antenatal care and to deliver at a healthcare facility.

*“There is no appropriate treatment at home. In these years, most men encourage their wives to go to the hospital or maybe taking the children themselves” [NFGD5].*

*“Men are the ones who encourage their wives to go for prenatal assistance and to go with a weak baby to the hospital” [MFGD3].*

Most of the men who were involved as participants in this study declared that they were involved in their family’s health and well-being, particularly in reference to MNCH.

*“When a woman is starting antenatal clinic they should all go together to receive counselling even if they have heard it before, but here they are advised again that you have to do this and that. Even if he parents are there, the man should take part as his family” [NFGD1].*

However, in terms of household level barriers, one of the issues that women reported was failure of their male partners in providing them with financial support for the purchase of the delivery items and transport costs for delivery. Women viewed this as a man’s responsibility. As one participant remarked,

*“Preparations of labour need cash so the husband is the one who is supposed to provide that cash” [N20*].

Without having the essential items required for the delivery, women were reluctant to deliver at the hospital as they would feel embarrassed and would fear being treated harshly by staff, or not receive treatment at all. Sometimes family members stepped in and purchased goods if husbands failed to do so. Sometimes the money was available, but used for other purposes, for example as reported by male participants, some men spent money on alcohol or on girlfriends and were therefore unable to cover the costs of their wives attending ANC and delivery at the hospital.

*“We can say that we men cheat a lot - that is why we reject our women… We may have things to support our wives, but we give these things to girlfriends leaving our wives starving. If we stop cheating and give a little of what we have found to the wives, there will be less deaths” [MFGD3]*.

Most participants voiced that women and men exercise an equal amount of power and authority within the family, but that each have different roles.

*“All people are one family so the woman can also have power as a man as one family” [NFGD1]*.

However, some women still needed permission from their husbands or other member of the family to attend clinic or hospital.

*“There are others [other problems] that men cause maybe because of power and the way they speak in the home that maybe everything that happens he should be told first; so maybe a child is sick and they cannot go to the hospital unless they tell the man; so the man has to make the decision that you can go to the hospital” [N7]*.

In terms of roles and responsibilities in the household it was expressed that women should look after the children in the daily care of the family, such as clothing, feeding and washing.

*“When it comes to breastfeeding and cooking for the child it is the role of a woman. The role of the man is to just go out and search for food” [NFGD2]*.

Men were expected to provide food or money for food, but also to encourage their wives to go to hospital when they or their children needed to, and arrange for this to happen. Men could encourage and persuade women to attend hospital and this was considered an important role for them to play. Men could also influence decisions over what food was given to children in his household; therefore, even though men might not have a direct responsibility in the preparing of food, he still needed to be informed on nutrition to make the right choices for his family.

The men who participated in the study were generally supportive of women attending hospitals:

*“No individual can stop his wife from going to the hospital… Not nowadays… That cannot happen… since they know that this is dangerous”* [N27].

However, men were not generally comfortable to be in the delivery room with their spouses during delivery.

*“This issue is very difficult for a man to go and watch how a woman is giving birth - as they have explained it can be difficult for a man to watch… Some of the women, because they are in pain during the time of labour, they say abusive language, and shout those words at a husband, so when coming back you can be concerned is that the way things are” [MFGD2]*.

### “Policy changes” to improve male involvement in MNCH

There is general support for the role of chiefs in MNCH. One participant in Nkhotakota suggested making it a law for men to attend ANC, whereas in an interview in Mduwa a participant noted that such a law exists and that it was the chiefs in Mchinji that passed this law in collaboration with the hospitals.

*“It is really happening, men are going to the hospital with their wives because there is a rule in place, that those that fail to go to the hospital, for example, from Nkangala, they will be paying maybe K5000.00” [MFG 3]*.

*“They may pay also in terms of goats. So they really go to the hospital together because they are afraid of that payment” [MFGD3].*

Community members generally accepted that these are ‘good’ laws and there is general support for a chief playing an active role in promoting MNCH attendance.

*“And so far the punishment they put looks to be working here because these days we are seeing husbands escorting their wives to the hospital” [M24].*

As one traditional leader informed us,

*“But nowadays we [traditional leaders] have been taught by the HSAs that if a woman is pregnant we should follow her up, we allow one woman who has children to go and ask if she has started going to ANC, if she doesn’t go, she will pay a goat we do not like that in the village” [N15].*

However, some participants noted that ANC staff sent women away if their husbands did not accompany them. In Mchinji, when men are being unsupportive of their wives attending ANC the chief can intervene and write a letter to the health staff to state that the woman can still receive ANC. As one religious leader reported,

*“They [health care workers] have come up with rules that every pregnant woman has to be accompanied by her husband during antenatal visits unless they present a letter from the chief justifying where the husband ran away; but every woman has to be accompanied by the husband” [M12].*

Traditional leaders are providing letters of support for married, unmarried and divorced pregnant women in order for them to access MNCH services. Interviews with some religious leaders, husbands and female users of MNCH services revealed the following;

*“Most men do not give their women to start pre-natal support when they are a month, two or three months. So when they go alone they are sent back because she is alone. When they go to pre-natal clinic at six months of pregnancy, they are told to bring a letter from the village headman that they may start prenatal support without their husband” [MFGD3]*.

*“Even antenatal when she is a student and also she was impregnated outside the wedlock chiefs are providing letters for them so that they can be assisted at the hospital early” [MFDG1].*

*“There were some pregnant women in here, one of them was from Ntcheu but they left husbands behind. How would they go to the hospital, when it is required that the husband and the wife should go together? So, I had to write a letter of recommendation for her and she was welcomed” [M30].*

For many of the men who participated in this study, feelings of discomfort and embarrassment were raised when discussing attending MNCH events. Some men reported feeling uncomfortable with participating in the health education component of the ANC visits because they felt shy and/or embarrassed about being asked to sing or dance with the women about reproductive health.

*“It is embarrassing to have to sing in a choir about MNCH because it may be your first time and you are 50 years old say and young ones who maybe are 14 years. They are like teasing you because they know us (laughter)” [MFGD3]*.

*“So men are like, so we should go there and clap hands as if we are dancing Chitelele while women are watching (laughter)?” [MFGD4]*.

One participant reported that men felt unwelcome by nurses and experienced long waiting times, but it was not clear whether these experiences were related to MNCH or to their own health needs.

Additionally, if they did attend, men reported that they felt uncomfortable sitting with women, interacting with young nurses and discussing things like sanitary pads with doctors. In some cases, men still did not feel that maternal and child health is their responsibility. As one participant observed:

*“We are not yet there, most men are not involved in issues of maternal child health, despite the information, but there is something that needs to be done to change the attitude of the male figures in the houses. It’s an issue of attitudes… Maybe they think that the issue of maternal health, the issue of bearing children, and the issue of child health are the issues of a woman. The message has been there for years, but we are not yet there” [N20]*.

Furthermore, one woman noted that sometimes men do not believe that attending the under-five clinic is a priority in the home. Some men feel that once a baby is born, husbands try to convince mothers that there is no need to attend a clinic if the baby is well by saying:

*“My wife I do not want my baby to go to under-five clinics let us do other things at home. So, what would you do as a wife because the baby belongs to the two of you it is not illegitimate because if it is illegitimate child you can do your own things. So if the husband says the money that he has is for drinking there is no money for soap it’s enough that we have a baby and he/she will grow” [FFGD].*

The fear of a going for a HIV test in case the result was positive (testing is done of both parents when ANC commences) was also raised as a barrier for men in attending ANC.

*“Not all of us will go along with the blood testing as we are afraid that my friends will isolate me (if found to be HIV)” [MFGD3]*.

Despite this unease, it was noted in one of the FGDs that even though they are mocked for going to ANC men feel they are doing the right thing.

*“We do accompany our women going to the ANC and there is no problem because people conclude that is how you show love to your family. Though people talk that you are charmed you only accept the talk and respond to them by saying that she is my wife whom I chose. I don’t stop escorting them at ANC because it is our choice” [MFGD2].*

### Community and health care provider initiatives for male involvement

Men in our study reported to be involved in initiatives, which foster communication about family planning. As one religious leader from Mkanda in Mchinji district noted,

*“… We have a group called Mai Mwana in our area and we meet them on the 22* ^*nd*^ *. 22* ^*nd*^ *is a day that we all meet like an umbrella meeting… all groups like CBOs, Mai Mwana, and from agriculture. In that meeting everybody is asked to give information they have to the people…. Mai Mwana teaches us on family planning to avoid over-population in our area” [RL1].*

A male participant provided an example of the collaboration between health service providers and community leaders, which was supported by funding from the UNICEF known as the “male championship model.”

*“Previously…we had male championship. There was a competition to see which part of the Traditional Authority (TA) is doing very fine on these issues. So, there was a completion on which areas is doing well on male championship, that is men who are also trying to disseminate information about family planning in all the TAs in Mchinji… And after that there was dissemination in Dowa, it was like a big event because there was an invitation of Ministry of Health’s personnel that presided over that function” [M5].*

Another man told us of the “male motivator” program which was supported by the Mai Mwana programme (translated from Chichewa to mean Mother and Child in English), in Mchinji, which trained about 10 men in each village to go and talk to other men on the importance of being involved in MNCH.

In Nkhotakota, a non-governmental official (NGO) reported that at community level they had established male only groups to encourage men to support women accessing MNCH services,

*“…at community level what we have done is that we have established also what we call: one man can. There are only male groups. They are mainly involved in male involvement influencing the information; the decision of men to make sure that there is support from men to makes rue that they support the women accessing these services” [N4].*

Another health care provider in the same district reported that collaborations between the community and health care providers have increased the involvement of men in MNCH issues.

*“The community involvement is high because people meet frequently. Village chiefs call for meetings with the people and nowadays you will find that the number of malnourished children has decreased. MNCH issues are discussed and, for instance, women are encouraged to take part in health issues, and men should also take part by taking their wives to the hospital. Moreover, if a child is sick they are advised to take him/her to the hospital. Nowadays men understand if the mother is not there they take the child to the hospital” [N6].*

The role of faith-based organizations, like churches, was mentioned as crucial to getting men involved in MNCH services. However, despite all these community and health care provider initiated activities, one government official from Nkhotakota district hospital observed that male involvement in MNCH issues is still a challenge.

*“...I do monthly assessment of how many men are attending those health talks…. We were surprised that over the past year from September 2013 to September 2014 we only had 4.6% of men attending these health talks” [N19].*

## Discussion

This study adds to the existing literature on male involvement in MNCH in Malawi by reflecting on the community system factors that encourage or impede men from supporting women’s utilisation of MNCH services. The involvement of men in the health of women and newborns around the time of childbirth – including but not limited to support for women during and after pregnancy, seeking skilled care for birth and complications, newborn care, nutrition and breastfeeding, family planning after childbirth and maternal health – has the potential to directly address gender influences on maternal and newborn health outcomes [43].

### Changing perceptions of traditional gender roles and power in decision-making?

The findings from our study echo findings from other qualitative studies on the perceived barriers and enablers to MNCH service utilisation. The main barriers to male involvement in MNCH identified in this study were at the household level, whereby husbands continue to retain control of the finances and decision-making in the home. In addition, men reported on the stigma men face when participating in activities thought of as “women’s business” [44]. Men reported to be shy to go to ANC as they were asked to sing or dance with the women and the fear of an HIV test being positive also emerged from the narratives of men. While most participants in our study expressed general agreement that men should accompany women to ANC, it is clear that in Malawi, dichotomous tensions exist between men being viewed as the providers and decision makers in their families and men being supportive of and engaging in women’s health seeking practices. It remains apparent that culturally defined gender roles and power relationships continue to serve as an obstacle to male involvement in MNCH.

However, our study also reveals positive strategies to involve “men as partners” and “men as positive agents of change” even if traditional constructions of masculinity and power remain unchallenged. In particular, getting men involved into peer groups, encouraging men to accompany their wives to ANC and participate in educative components of ANC services deliberately help target gender relations in order to challenge patriarchal structures that reinforce pre-existing roles and norms surrounding masculinity. Our findings reveal the community and health care provider efforts to transform gender relations, norms and structures in order to improve MNCH outcomes in Mchinji and Nkhotakota. We also note that some of the strategies employed can produce the opposite effect, if not carefully initiated. What is promising about these strategies is the intention to change the behaviour of men in the community.

### “Policy-changes” and community-based initiatives to engage men

Community leaders play a significant role in influencing health service use among rural communities in sub-Saharan Africa. Our findings reveal community leaders in our study (all who were men), played an active role in the promotion of knowledge on the MNCH services provided and the role of men in supporting women in utilising these services. As mentioned earlier, in Malawi, the Safe Motherhood program has empowered traditional leaders to make sure that all pregnant women deliver at a health facility. In Malawi, the engagement of chiefs has provided an opportunity for involving men in MNCH, and the narratives of the men we interviewed revealed that they were likely to follow the advice when a respected member of the community delivers it. As Kululanga et al., observe, “Involvement of chiefs and village headmen/women in public health interventions demonstrate the importance of the issue and therefore motivate greater participation of other community members” [45]. In addition, the authority and respect that traditional leaders command offer a vehicle “through which social and cultural changes can be realized” [45, 46]. Moreover, chiefs, in Malawi, have instituted bylaws that penalize men if they do not accompany women to ANC (“go to scale”) and are present at the delivery. Tanzania, Zimbabwe and Malawi have reported on the perceived effectiveness of the implementation of bylaws initiated by community leaders on engaging community members for better MNCH outcomes [47]. Despite the deployment of punitive tactics such as the imposition of fines, chiefs provide the leadership that may be necessary to create an environment that encourages the use of MNCH services. To this extent, traditional leaders have collaborated with the formal health care system to promote the involvement of men in MNCH service utilization. In short, traditional leaders are community level enablers and can strengthen community systems to support the utilisation of MNCH services; however, we note that this practice should be undertaken in a supportive (rather than coercive) manner so as to avoid counter-productive health seeking behavioural practices.

Another strategy to engage men involved the health care providers in collaboration with community leaders. The “male championship” and Mai Mwana programmes in Mchinji were mentioned by participants as important initiatives for involving men to improve the health of mothers and infants. Both programmes are anchored on the use of peer educators. The use of peer educators (male motivators) was perceived as an effective community-based strategy to include and involve men by targeting men with messages focused on the benefits of utilizing MNCH services. From the narrative accounts provided by participants in Mchinji, it became clear that such activities helped challenge traditional gender norms. Our findings are similar to observations made by Kadomoto et al., in the Philippines whereby it was reported that teaching men about maternal and child health improved men’s involvement in the services, but also enabled the same men to become educators of other men in the community [46]. In essence, peer-educators are useful for influencing men and encouraging greater participation in MNCH services.

### Health facility initiatives to engage men

The use of incentives to encourage male involvement is employed as a strategy to reward positive behaviour to improve MNCH. The use of incentives is what was reported in our study from participants in Mchinji district. In fact, a similar finding was revealed in a study done by Kululanga et al., in Mwanza district, where participants at Mwanza district hospital described the same type of competition organized by UNICEF in 2008 [29]. In addition, incentives also come in the form of receiving priority care at health centres and hospitals. Our study also confirmed that women who are accompanied with their husbands to ANC clinic are assisted and attended to first. A current practice at hospitals and health centres in Malawi is to incentivise women who come with their husbands by providing the couple with priority services. Even promotional messages are displayed to encourage and reward partners that attend ANC together. In an urban hospital in Malawi, for example, a caption under a picture in the ANC facility reads as follows: “Go to the hospital during the first three months together with your husband” [48] A husband accompanying his pregnant spouse rewards the couple with priority service and this type of treatment is not unique to Malawi. In Kenya, for example, health staff promoted the involvement of husbands in ANC through giving preferential treatment and a free shawl for their child if the husband attended ANC with his wife [48]. However, as Kululanga et al. warn, the use of incentives for couples attending ANC together can result in stigmatisation and unfair treatment of women who are unsupported and/or must choose to attend without a male partner [46]. Ultimately though, despite this concern, we acknowledge the potential role that incentives can play in encouraging behaviour change in men.

## Study limitations

Our study participants were mainly from the two districts we selected as case studies and our findings reflect the opinions of the individuals we interviewed in these two districts. In order to further contribute to a holistic understanding on the community system barriers and enablers to MNCH service utilization it may have been useful to interview key informants such as policy makers, NGO officials, technocrats and donor partners as their opinions may have further enriched our findings.

## Conclusion

Traditional notions of men as decision makers and socio-cultural views on maternal health continue to present challenges to male involvement in MNCH programs. The main obstacles to male involvement do not only centre on the traditional constructions on masculinity and power. Overall, what is critical to catalysing social change are approaches that view men as partners or view men as agents of positive change. The informal bylaws of traditional leaders, health facility initiatives and the health care provider strategies provide a range of opportunities to engage and involve men in supporting women’s use of MNCH services. However, the use of coercive or punitive measures as well as competitions to drive men to participate in what for them can be a threatening space, is not the best approach to creating deep and meaningful behaviour change. At a programmatic and content level, community-led initiatives need to use entertainment for example, drama and plays, that are culturally acceptable to both men and women, during community meetings to address gender-related barriers and facilitators of MNCH service utilisation. Health care provider initiatives need to be sensitive and mindful of gender roles and relations by, for example, creating gender inclusive programs and spaces that aim at reducing perceptions of barriers to male involvement in MNCH services so that programs and spaces that are aimed at involving men are designed to welcome men as full partners in the overall goals for improving maternal, neonatal and child health outcomes.

## Additional files

Additional file 1: Appendix 1. In-depth interview guide. (DOCX 113 kb)

Additional file 2: Appendix 2. In-depth interview guide. (DOCX 106 kb)

Additional file 3: Appendix 3. In-depth interview guide. (DOCX 103 kb)

Additional file 4: Appendix 4. In-depth interview guide. (DOCX 126 kb)

Additional file 5: Appendix 5. Focus Group Discussion guide. (DOCX 135 kb)

## Abbreviations

ANC: Antenatal care
COMREC: College of Medicine Research and Ethics Committee
COSYST-MNCH: Community systems strengthening for equitable maternal, newborn and child health
FGDs: Focus group discussions
HSAs: Health surveillance assistants
IDIs: In-depth interviews
MNCH: Maternal, newborn and child health
NGOs: Non-governmental organizations
PMTCT: Prevention of mother-to-child transmission
T/A: Traditional authority
TBAs: Traditional birth attendants
