# Supplementary material for: Changing times? Gender roles and relationships in maternal, newborn and child health in Malawi
Source: BMC Pregnancy Childbirth. 2017 Sep 25;17:321. doi: 10.1186/s12884-017-1523-1 (PMC5613316; doi:10.1186/s12884-017-1523-1)
Supplement: Supplementary file 1 — Appendix 1. In-depth interview guide. (DOCX 113 kb) [file 12884_2017_1523_MOESM1_ESM.docx]

**Additional file 1: Appendix 1. Focus Group Discussion Interview Guide for Women**

**Key actors to be interviewed include women:**

- - who are using MNCH services
  - who are not using MNCH services
  - those affected by disease or vulnerabilities

1. What MNCH services exist in the community?
2. Are MNCH services accessible to you?
3. What are the issues that prevent you from attending the clinic for ANC/delivery/PNC and immunisations?
4. How long does it take you to get to the clinic? How do you travel there? Are there times of the year when you cannot access the clinic?
5. What are the main areas of activity and specific services provided in the community? (Probe for different sectors)
6. What is the scale and reach of activities?
7. Have activities evolved over time (e.g. in response to external actors, availability of funding of local need)?
8. Has geographical coverage evolved over time?
9. Who is the target audience for different services? (e.g. the whole of the community or specific target groups
10. Do the community based activities receive external support? If so, what are the sources of external support?
11. Are community services acceptable? Is there an awareness of available services? Does this vary by sub-group within the community?
12. Are there specific community services that assist/prevent mothers using MNCH services?
13. Does the community help you to cope with illness, poverty and negative events? How?
14. Are there factors that impede access to MNCH services/care at the community and household level?
15. Have you been involved in decisions about how MNCH services are delivered?
16. Is there a good relationship between the clinic and the community? How does this work?
17. Are there HSAs in this village? How do you perceive them? Do they work well with the clinic?
18. Do you perceive any risks in not attending the clinic for ANC, delivery postnatal care etc.? What are these? Why do you attend/not attend?
19. Does the community play a role in influencing your decision about whether you attend for ANC, delivery, PNC, immunizations, childhood illness?
20. What are the risks/benefits to attending the clinic for ANC/delivery/PNC/immunizations, childhood illness?
21. Do you/the community feel that you have ownership over decision making for MNCH in the community?
