# Supplementary material for: Changing times? Gender roles and relationships in maternal, newborn and child health in Malawi
Source: BMC Pregnancy Childbirth. 2017 Sep 25;17:321. doi: 10.1186/s12884-017-1523-1 (PMC5613316; doi:10.1186/s12884-017-1523-1)
Supplement: Supplementary file 2 — Appendix 2. In-depth interview guide. (DOCX 106 kb) [file 12884_2017_1523_MOESM2_ESM.docx]

**Additional file 2: Appendix 2. Focus Group Discussion Interview Guide for Caregivers**

**Key actors to interview include household members, e.g., husbands, grandmothers, mothers in-law:**

1. What MNCH services exist in the community?
2. Are MNCH services accessible to you?
3. Who is involved in providing outreach services?
4. How long does it take you to get to the clinic? How do you travel there? Are there times of the year when you cannot access the clinic?
5. What are the main areas of activity and specific services provided in the community? (Probe for different sectors)
6. What is the scale and reach of activities?
7. Have activities evolved over time (e.g. in response to external actors, availability of funding of local need)?
8. Has geographical coverage evolved over time?
9. Who is the target audience for different services? (e.g. the whole of the community or specific target groups)
10. Do the community based activities receive external support? If so, what are the sources of external support?
11. Are community services acceptable? Is there an awareness of available services? Does this vary by sub-group within the community?
12. Are there specific community services that assist/prevent mothers using MNCH services?
13. Do you work with other members of the community to solve problems/ improve health? How?
14. Does the community have common rules, norms and sanctions?
15. Is there connectedness between and among networks and groups to help bridge different points of view?
16. In what ways (if at all) do gender and other power relations impact uptake of MNCH services?
17. Does the status quo of the community assist/impede MNCH service uptake?
18. Describe what matters to people in the community:
    1. Issues in the community that people care about (e.g. safety, education, housing, health)
    2. How important these issues are to the community (e.g. perceived importance, consequences for the community)
19. What, in your opinion**,** does the community care about and what does it ignore? What are the norms for interaction among those with different opinions or different backgrounds?
