# Supplementary material for: Changing times? Gender roles and relationships in maternal, newborn and child health in Malawi
Source: BMC Pregnancy Childbirth. 2017 Sep 25;17:321. doi: 10.1186/s12884-017-1523-1 (PMC5613316; doi:10.1186/s12884-017-1523-1)
Supplement: Supplementary file 3 — Appendix 3. In-depth interview guide. (DOCX 103 kb) [file 12884_2017_1523_MOESM3_ESM.docx]

**Additional file 3: Appendix 3. In-depth Interview Question Guide for Community leaders**

**Key actors to interview include: village chiefs, tribal chiefs, village leaders, religious leaders**

Note to interviewer: Remember to gather a few socio-demographic characteristics of the respondent e.g., name, age, marital status, parity.

1. Who are the leaders in the community? (formal and informal)
2. Are the elected/self appointed? From a faith community?
3. What formal/informal powers does the tribal/village chief hold? How do they work to support/undermine MNCH services, both formal and informal?
4. What types of networks/groups exist in the community? (Probe for specific sectors, actors and groupings including MNCH)
5. Do they link with one another?
6. Does the Village have a Village Health Committee? *Village Health Committees:*
   1. What are the functions of the VHC? (e.g., support the management of CHWs, define HW functions, coordinate services and programmes in each CHW area etc.)
   2. Is the position of the VHC within the village ever contested? Why?
7. Do traditional healers play an important role in health in the community? If yes, what role do traditional healers play in the community? In your opinion, do traditional healers enable or constrain the utilisation of formal health services?
8. Do religious leaders have an important role to play in the health of the community? If yes, what role do they play? In your opinion do religious leaders enable or constrain the utilisation of formal health services?
9. In your opinion, why do people seek care from the different types of health care providers e.g., orthodox and traditional.
10. Are community leaders considered partners in the planning of health services?
11. The long-term history of the community can tell you about community traditions. In your opinion, what has the community been proud of, and what would residents prefer not to talk about, for example, factions within the community, the past, the present?
12. How do the people in the community relate to one another on a daily basis; how are problems resolved.
