# Supplementary material for: Changing times? Gender roles and relationships in maternal, newborn and child health in Malawi
Source: BMC Pregnancy Childbirth. 2017 Sep 25;17:321. doi: 10.1186/s12884-017-1523-1 (PMC5613316; doi:10.1186/s12884-017-1523-1)
Supplement: Supplementary file 4 — Appendix 4. In-depth interview guide. (DOCX 126 kb) [file 12884_2017_1523_MOESM4_ESM.docx]

**Additional file 4: Appendix 4. In-depth Interview Question Guide for Community Organizations**

**Key actors to interview include: Senior representatives of NGOs, government departments, district health facilities/ Representatives from community groups/organisations**

Note to interviewer: Remember to gather a few socio-demographic characteristics of the respondent e.g., name, age, marital status, parity.

1. What is the main focus of the organisation/group/initiative?
2. Does it have paid staff/volunteers?
3. What are the funding channels?
4. Was the initiative initiated by actors within the community (e.g. individuals, existing community structures or groups) or external actors?
5. How are the organisations’ activities linked to and aligned with government services (e.g. local government, health facilities, social welfare services, and health, education and justice departments)?
6. Are referral systems in place between the community-based organisations and service providers?
7. What partnership arrangements are in place (e.g. formal partnerships and MoUs, informal partnerships, collaborations or shared networks)?
8. How do actors in the community participate in coordination mechanisms, networks, umbrella organisations or coalitions?
9. Which actors are driving partnerships and collaborations?
10. What local communication/media approaches exist at community level? (e.g. signs, local radio, megaphones and local theatre) specifically for MNCH services?
11. What alert systems are used within the community to call members to a meeting to discuss community issues?
12. What community-based information systems exist? Do they allow the community to understand the epidemiology of their own community settings and prioritise solutions**?**
13. Are mobile technologies used?
14. Does the community receive feedback from the District on health data?
15. Does the community use data for planning services at the district level?
16. What monitoring and evaluation is undertaken in the community and how effective is it?
17. Are there any peer support groups in the community?
    - What types exist? (mother to mother, women’s, grandmothers, PLWHA, community associations)
    - Why were they established?
18. Does the private health sector play a role in the community? If yes, in what role does the private health sector play in the community? (Probe for different types of organisations and different roles)
19. Do you think faith-based organizations have a role to play in promoting community health?
20. If yes, what role do FBOs play in the community? If no, what role do you think FBOs should play? (Probe for positive/negative roles).
21. Do cross-sectoral committees exist to address determinants of MNCH?
22. Who is involved in providing outreach services?
23. How are they implemented? How do they link with the formal system?
24. Are they facility directed (community plays the role of arranging logistics, notifying other community members, and supporting health education activities) OR
25. Community directed? (community plays an extensive role in the design of the activity, its implementation and monitoring of the outcome)
26. Are community events used to increase community knowledge and demand of MNCH services? How?
