# Supplementary material for: Changing times? Gender roles and relationships in maternal, newborn and child health in Malawi
Source: BMC Pregnancy Childbirth. 2017 Sep 25;17:321. doi: 10.1186/s12884-017-1523-1 (PMC5613316; doi:10.1186/s12884-017-1523-1)
Supplement: Supplementary file 5 — Appendix 5. Focus Group Discussion guide. (DOCX 135 kb) [file 12884_2017_1523_MOESM5_ESM.docx]

**Additional file 5: Appendix 5. In-depth Interview Question Guide for Representatives of service users of Formal/Informal health related services/Community Health Workers (CWH)**

**Key actors to interview include: Frontline health workers based in the community, e.g. nurses, health surveillance assistants, clinical officers, traditional birth attendants**

Note to interviewer: Remember to gather a few socio-demographic characteristics of the respondent e.g., name, age, marital status, parity.

1. Which organization do you work for/represent?
2. How long have you worked in the field of health, more specifically, in the area of MNCH?
3. Are there linkages between the health system and the community?
4. Do community/health services partnerships exist for MNCH services? What are they?
5. Is there community participation in decision making around local MNCH issues?
6. Are skilled community health workers in place?
7. Are the numbers and training of HSAs sufficient?
8. Is there a referral system in place that adequately supports facilities providing primary care for mothers and children?
9. Are there factors that impede access to MNCH services/care at the community and household level?
10. Can you tell me if there are CHWs in the community? Have you had contact with them? For what purpose?
11. Do they encourage links with the health facility/hospital?
12. Are the CHWs specialised or generic? What services do they provide to women and children?
13. Does the CHW have a support group or is (s)he linked to a health committee?
14. Is the role of the CHW and frontline health workers clearly defined?
15. Does the community trust the CHWs?
16. Are there efforts to leverage community resources to promote MNCH?
17. To what degree is the community involved in promoting MNCH (e.g., community leads, actively participates, supports, is simply informed, is co-opted?)
18. Who leads the various community initiatives (e.g., initiated and led by community, initiated and led by external actors, or initiated by external actors and led by the community, volunteers or paid staff)?
19. What activities or interventions are the community involved in (e.g., identifying problems, needs, and priorities; planning and design; implementation and service delivery; M&E)?
20. Has the involvement of the community been sustained over time (e.g., ongoing relationship, occasionally involved, or once-off consultation)? Ask for the various initiatives.
21. Who is involved in providing outreach MNCH services in the community?
22. How are they implemented? How do they link with the formal system?
23. Are they facility directed (community plays the role of arranging logistics, notifying other community members, and supporting health education activities)? Or are they community directed? (community plays an extensive role in the design of the activity, its implementation and monitoring of the outcome)
24. Are community events used to increase community knowledge and demand of MNCH services? How?
